# Critical evaluation of *KCNJ3* gene product detection in human breast cancer: mRNA in situ hybridisation is superior to immunohistochemistry

**DOI:** 10.1136/jclinpath-2016-203798

**Published:** 2016-10-03

**Authors:** Sarah Kammerer, Stephan Wenzel Jahn, Elke Winter, Sylvia Eidenhammer, Simin Rezania, Peter Regitnig, Martin Pichler, Wolfgang Schreibmayer, Thomas Bauernhofer

**Affiliations:** 1 Molecular Physiology Group, Institute of Biophysics, Medical University of Graz, Graz, Austria; 2 Research Unit on Ion Channels and Cancer Biology, Medical University of Graz, Graz, Austria; 3 Institute of Pathology, Medical University of Graz, Graz, Austria; 4 Division of Oncology, Department of Internal Medicine, Medical University of Graz, Graz, Austria

**Keywords:** BREAST CANCER, IMMUNOHISTOCHEMISTRY, IN SITU HYBRIDISATION, ANTIBODIES, METHODOLOGY

## Abstract

Increased expression levels of *KCNJ3* have been correlated with lymph node metastases and poor prognosis in patients with breast cancer, suggesting a prognostic role of *KCNJ3*. We aimed to establish protocols for the detection of *KCNJ3* in formalin-fixed, paraffin-embedded (FFPE) breast cancer tissue. Several antibodies were tested for sensitivity and specificity by western blot, followed by optimisation of the immunohistochemistry (IHC) procedure and establishment of *KCNJ3* mRNA in situ hybridisation (ISH). Methods were validated by processing 15 FFPE breast cancer samples for which microarray data were available. Spearman's rank correlation analysis resulted in borderline significant correlation for IHC versus ISH (*r_S_:* 0.625; *p*<0.05) and IHC versus microarray (*r_S_:* 0.668; *p*<0.01), but in significant correlation for ISH versus microarray (*r_S_:* 0.861; *p*<0.001). The ISH method was superior to IHC, regarding robustness, sensitivity and specificity and will aid to further study expression levels of *KCNJ3* in both malignant and physiological conditions.

## Introduction

*KCNJ3* encodes for G-protein activated inward rectifier potassium channel 1 (GIRK1; synonyms: KGA, Kir3.1). GIRKs are G-protein effectors that regulate cellular excitability and activity via neurotransmitters and hormones. Physiological roles of GIRKs include, among others, regulation of heartbeat, learning and memory functions.^1^ Most importantly, several lines of evidence demonstrate a correlation of increased *KCNJ3* expression levels, breast cancer progression and patient’s prognosis. Stringer *et al*^2^ observed increased levels of *KCNJ3* mRNA in primary invasive breast carcinomas when compared with corresponding normal breast tissue and found *KCNJ3* mRNA levels positively correlating with the amount of metastatic lymph nodes. A study by Brevet *et al*^3^ supported these results by showing a correlation between GIRK1 protein expression and lymph node metastases as well as reduced overall survival of patients with tumours displaying high GIRK1 expression. The relevance of *KCNJ3* in breast cancer is further underscored by Rezania *et al*,^4^ who demonstrated increased motility, invasiveness and angiogenesis of *KCNJ3* overexpressing MCF-7 breast cancer cells compared with controls. Ko *et al*^5^ found *KCNJ3* to be downregulated in p53 mutant breast cancer samples and upregulated in oestrogen receptor (ER) positive tumours. Our own findings^6^ also show a strong link between *KCNJ3* expression and ER-positive tumour samples. Taken this evidence together, it seems worthwhile to study *KCNJ3* expression in invasive breast carcinoma to validate it as a new prognostic biomarker for this disease. In order to pursue this goal, we aimed to establish methods for the detection of *KCNJ3* gene products in formalin-fixed, paraffin-embedded (FFPE) breast cancer samples. Many commercially available antibodies are insufficiently tested in terms of sensitivity and specificity, which in turn can lead to misleading results, and also others experienced difficulties with anti-GIRK1 antibodies.^7^ Therefore, the aim of this study was to (a) critically test a panel of anti-GIRK1 antibodies for sensitivity and specificity, (b) to systematically optimise experimental immunohistochemistry (IHC) conditions, (c) to establish RNA in situ hybridisation (ISH) protocol as an antibody-independent and microarray-independent method for the detection of *KCNJ3* mRNA in tumour samples, and (d) to determine the correlation of GIRK1 protein (IHC) with *KCNJ3* mRNA (microarray and ISH) expression in human breast cancer in order to identify the best *KCNJ3* detection method.

## Materials and methods

### Anti-GIRK1 antibodies

Ab#1: mouse monoclonal antibody from Abcam (#Ab11924); Ab#2: rabbit polyclonal antibody directed against the C-terminus of GIRK1 (generated by Kurt Schmidt, Institute of Pharmaceutical Sciences, University of Graz, Austria; described in ref. 8); Ab#3: rabbit polyclonal antibody directed against the N-terminus (N-T) of GIRK1 (generated by Kurt Schmidt); Ab#4: polyclonal goat antibody from Santa Cruz (#Sc-16131); Ab#5: rabbit polyclonal antibody from Alomone (#APC-005) and Ab#6: mouse monoclonal antibody from Alomone (#ALM-031). Sheep anti-rabbit/horseradish peroxidase (HRP), sheep anti-mouse/HRP (both kindly provided by Amir-Hassan Zarnani, Avicenna Research Institute, Tehran, Iran; both 1:10 000 dilution) and donkey anti-goat IgG-HRP antibodies (Santa Cruz Biotechnology, #Sc-2020; 1:5000 dilution) served as secondary antibodies.

### Cell culture

MCF-7 wild-type or stably overexpressing *KCNJ3* and HEK-293 cells (both from ATCC) were cultured as described.^4^ ^8^ HL-1 cells were purchased from William C. Claycomb and maintained as described.^9^ Cells were kept in a humidified atmosphere at 37°C and 5% CO_2_. Mycoplasma tests were negative, and short tandem repeat profiling proofed the cell lines to be authentic.

### Patient samples

FFPE tissue samples of ER-positive primary invasive breast carcinomas were selected from a series of previously published breast cancers,^10^ for which microarray analysis (Affymetrix, GEO accession GSE17705) had been performed. Out of these, 15 tumour samples representing carcinomas with highest (n=5), intermediate (n=5) and lowest (n=5) *KCNJ3* microarray expression levels were retrieved from the biobank of the Medical University of Graz. ER-positive samples were chosen, since a strong correlation between *KCNJ3* expression and ER-positive breast cancer has been reported.^5^ ^6^ All cases were annotated with detailed clinical and pathological information (see table 1 for patient characteristics). The use of the patient samples including the clinical data was approved by the ethics committee of the Medical University of Graz (24-081 ex 11/12).

**Table 1:** Patient characteristics and *KCNJ3* expression levels as determined by different methods

	Clinicopathological patient characteristics							Results of *KCNJ3* expression		
#	AAD	Grade	pT	LN	pM	ER	PR	Microarray	IHC	RNA ISH
01	78	2	2	3/20	−	+	+	11.407	3	33.33
02	60	2	2	5/20	−	+	+	9.417	3	10.11
03	56	2	2	3/23	−	+	+	9.393	3	9.01
04	71	2	2	0/29	−	+	+	9.109	2	2.69
05	68	3	2	0/18	−	+	−	8.917	1	0.33
06	68	2	1	11/18	−	+	+	8.239	3	1.28
07	43	2	3	2/8	−	+	−	8.207	1	2.10
08	50	1	4	0/11	−	+	−	8.061	3	0.28
09	70	3	2	0/17	+	+	+	8.042	2	1.53
10	63	2	2	0/14	−	+	−	7.919	0	0.16
11	62	3	2	9/27	+	+	+	7.205	1	0.07
12	50	1	1	3/16	−	+	+	7.174	1	0.28
13	63	2	1	0/0	+	+	+	6.918	1	1.26
14	53	2	4	16/19	−	+	+	6.740	2	0.07
15	70	2	2	3/14	−	+	−	5.656	0	0.07

#, patient sample number; −, negative; +, positive; AAD, age at diagnosis; ER, oestrogen receptor; IHC, immunohistochemical score of *KCNJ3* protein expression; LN, lymph nodes (positive/total examined); Microarray, log2 intensities of *KCNJ3* expression; pM, distant metastasis status; PR, progesterone receptor; pT, tumour size staging; RNA ISH, *KCNJ3* RNA in situ hybridisation results as spots/cell.

### Immunohistochemistry

FFPE sections of patient samples were mounted on glass slides together with formalin-fixed, agarose embedded HL-1 and HEK-293 cells which served as positive and negative on-slide controls.^11^ ^12^ Staining conditions were optimised by systematic variation of staining conditions regarding heat-induced epitope retrieval, washing solutions and antibody dilution. A detailed description of the IHC protocol is given in online supplementary file 1. Cytoplasmic staining intensity in tumour tissue was scored by two independent investigators (SWJ and TB) using a semiquantitative four-tiered score with 0–3 corresponding to no staining (0), weak (1), intermediate (2) and strong (3) staining. Incubation without primary antibody served as additional negative control.

10.1136/jclinpath-2016-203798.supp1 Supplementary file

### RNA in situ hybridisation

Sections of FFPE tissue (thickness 4 µm) were mounted on Superfrost Plus coated slides (Thermo Scientific) and invasive tumour areas were selected by trimming away peritumoural tissue devoid of cancer. The slides were processed according to manufacturer's instructions for the RNAscope 2.0 High Definition—BROWN kit (ACD). Three sections of each sample were stained with different probes: the *KCNJ3* probe (#Hs-KCNJ3-tv1tv2), the negative control probe *DapB* (#310043) and the positive control probe *POLR2A* (#310451). For image analysis, a representative tumour region was selected for each tumour, and z-stacks comprising 10 images were captured at 40× magnification using a Zeiss Oberver.Z1 inverted microscope. Multiple adjacent single images (3×3 tiles) were acquired and aligned using the MosaiX module of the AxioVision software (Zeiss). Image sequences were stacked, and the SpotStudio software from ACD was used for detection of single cells, detection of spots and clusters and calculation of estimated number of spots per cell (see online supplementary file 2, eg, of image analysis). *DapB* and *POLR2A* probes served as technical quality controls that needed to fulfil the cut-off criteria (≤0.5 spots/cell for negative controls; ≥2.5 spots/cell for positive controls) in order to ensure technical specificity of the probes and to detect samples with highly degraded RNA.

10.1136/jclinpath-2016-203798.supp2 Supplementary file

### Statistical analysis

Spearman's rank correlation analysis was performed to correlate the results of the different *KCNJ3* detection methods. Analyses were performed using the SigmaPlot/SigmaStat V.12.5 software (Systat Software). Results with *p*<0.05 were considered statistically significant.

## Results

### Screening of different anti-GIRK1 antibodies for specificity and sensitivity

**Figure 1:**
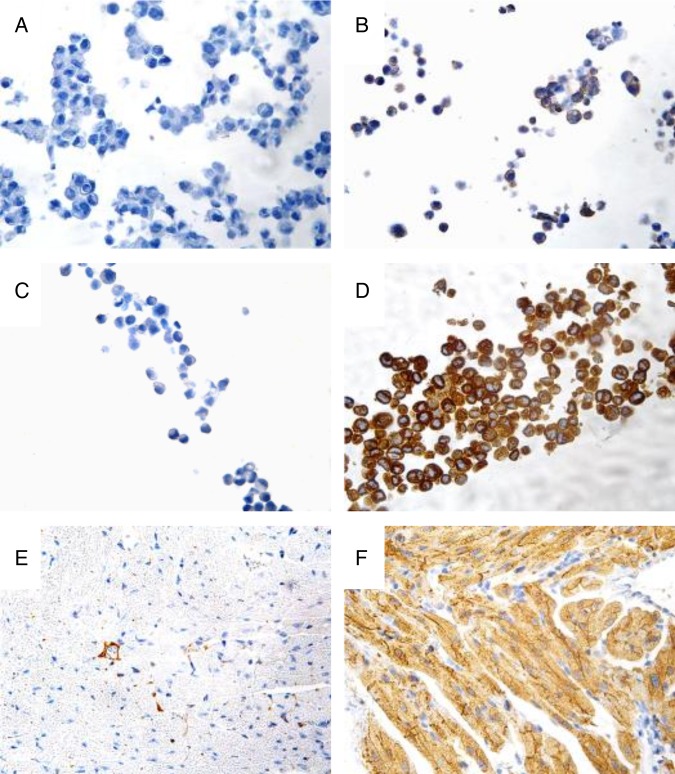
Performance of Ab#1 for immunohistochemistry on cell lines and formalin-fixed, paraffin-embedded mouse heart. (A) GIRK1 immunohistochemistry on formalin-fixed, agarose-embedded HEK-293 cells (negative control), (B) HL-1 cells (positive control), (C) MCF-7 cells and (D) MCF-7 cells overexpressing *KCNJ3*. (E) GIRK1 immunohistochemistry on FFPE mouse ventricle and (F) atrium. Micrographs were taken at 40× magnification.

Specificity and sensitivity of the different anti-GIRK1 antibodies were first assessed by Western blot analysis as described in online supplementary file 3. In summary, Western blots recommended Ab#1 for best sensitivity and specificity, and it was therefore further tested for its suitability in IHC on formalin-fixed, agarose-embedded cell pellets and FFPE mouse tissue (figure 1). As expected, staining was absent in HEK-293 (figure 1A) and positive in HL-1 cells (figure 1B). Wild-type breast cancer MCF-7 cells were negative (figure 1C), in contrast to strong positivity of MCF-7 cells overexpressing *KCNJ3* (figure 1D). Tissue sections from mouse atrium and ventricle served as additional positive and negative controls^13^ with expected negativity of GIRK1 staining in the ventricle in contrast to strong positivity of the atrial myocardium (figures 1E, F). In summary, Ab#1 yielded satisfactory results regarding specificity and sensitivity, and the protocol was further optimised for archived human FFPE breast cancer samples using patient sample #2 with high *KCNJ3* mRNA expression levels (according to microarray data; table 1) as biological positive control (see online supplementary file 4 for details). The combination of heat-induced epitope retrieval at pH 9 (microwave), usage of Dako wash buffer and an antibody dilution of 1:50 of Ab#1 lead to best staining results with highest specificity and lowest background. This protocol was therefore used as standard protocol for the staining of the remaining patient samples.

10.1136/jclinpath-2016-203798.supp3 Supplementary file

10.1136/jclinpath-2016-203798.supp4 Supplementary file

### IHC staining results of FFPE breast cancer samples

**Figure 2:**
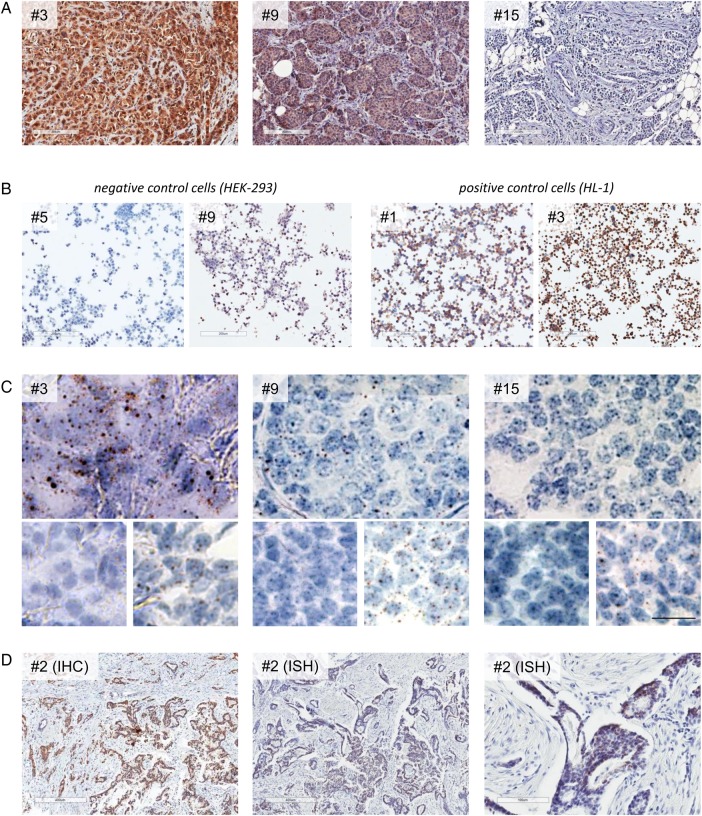
Comparison of *KCNJ3* protein (GIRK1) and mRNA expression in formalin-fixed, paraffin-embedded samples of patients with human breast cancer. (A) GIRK1 immunohistochemistry (IHC) using the optimised protocol for Ab#1. Generic micrographs for patients with high (#3), intermediate (#9) and low (#15) GIRK1 protein expression are shown. (B) Two examples of on-slide negative control cells (HEK-293 on slides of samples #5 and #9) and positive control cells (HL-1 on slides of samples #1 and #3). (C) *KCNJ3* RNA in situ hybridisation (ISH) results of the same samples as used in (A). *Top panel*: *KCNJ3* probe. *Lower panel left*: *DapB* probe (negative control); *right*: *POLR2A* probe (positive control). Scale bar: 20 µm; all images shown at identical magnification. (D) Positive *KCNJ3* signals are present in tumour cells but not in non-neoplastic cells. *Left:* GIRK1 IHC of patient sample #2; *middle*: *KCNJ3* RNA ISH of patient sample #2; *right*: detail of middle image.

**Figure 3:**
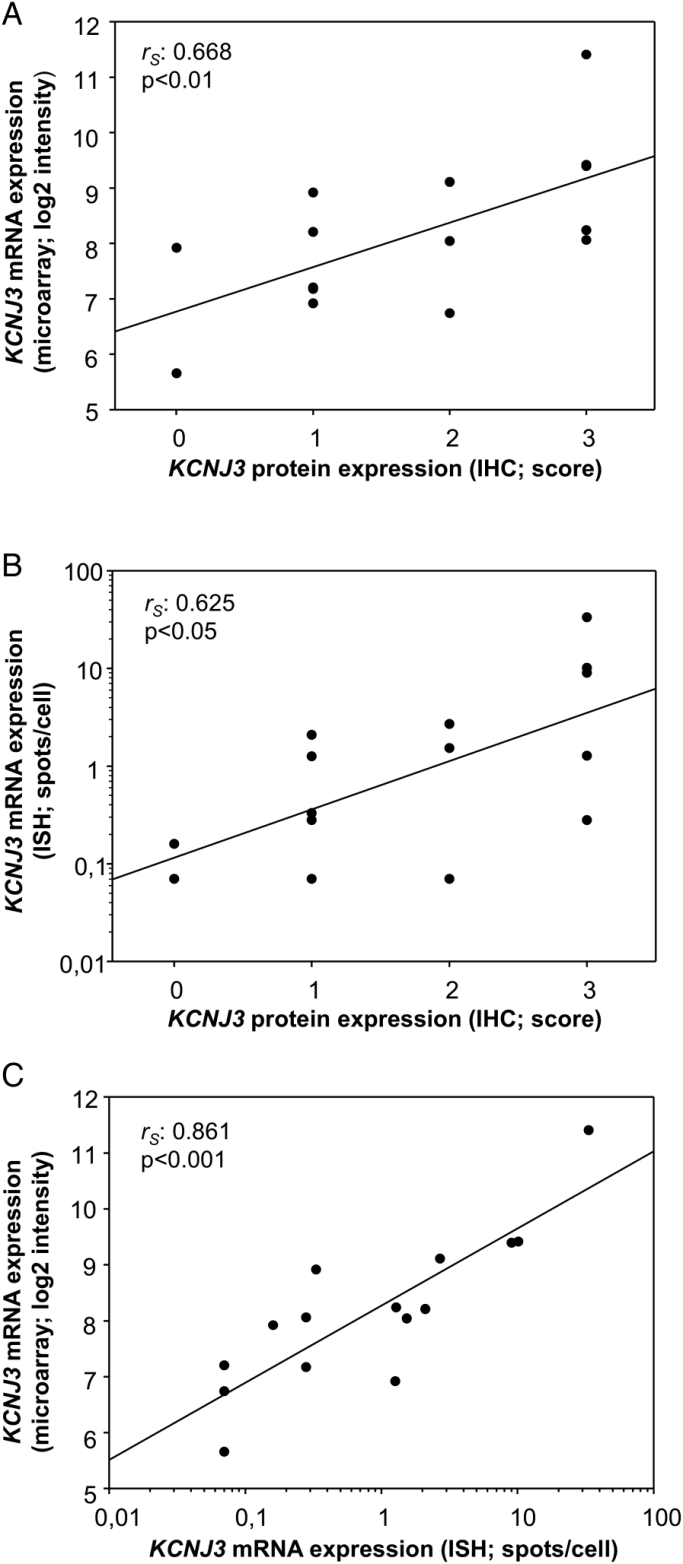
Correlation of *KCNJ3* gene product expression as assessed by different methods. (A) Scatter plot of *KCNJ3* expression levels in 15 patients with breast cancer as assessed by microarray (log2 intensity) and immunohistochemistry (IHC) score. (B) RNA in situ hybridisation (ISH; spots/cell) versus IHC (score) is plotted. (C) Log2 intensity of microarray versus RNA ISH (spots/cell) is plotted. Spearman's rank correlation coefficients (*r_S_*) and *p* values are given for each plot.

Subsequently, GIRK1 IHC was performed on 15 patient samples with different levels of *KCNJ3* mRNA expression as assessed by microarray analysis (see table 1 for patient characteristics, microarray data and staining results). Representative images of patient samples with high, intermediate and low *KCNJ3* expression are displayed in figure 2A. On each IHC slide, formalin-fixed, agarose-embedded HEK-293 and HL-1 cells were mounted as on-slide controls. Assessment of these controls revealed that antibody staining was not consistent, even in same runs, as shown exemplarily in figure 2B. However, positive staining was found in tumour cells only, but not in tumour stroma, inflammatory cells or normal structures including mammary ducts (figure 2D, left). IHC staining results correlated significantly with the microarray data (*p*<0.01), with a correlation coefficient of *r_S_*=0.668 (figure 3A).

### RNA ISH staining results of FFPE breast cancer samples

Further, we aimed to establish a protocol for RNA ISH in order to quantitate *KCNJ3* mRNA directly in FFPE sections and to correlate the results with microarray data and IHC staining. To achieve maximum technical concordance between microarray and RNA ISH data, the specific *KCNJ3* probes for ISH covered RNA regions identical to the Affymetrix microarray assay. Technical negative and positive controls all met the given cut-off criteria, and samples were therefore regarded as successfully stained. In addition, sample #6 was used as biological positive control in each run (see online supplementary file 5). Results are given in table 1, and representative images of patient samples and their respective controls are displayed in figure 2C. In concordance with the IHC results, *KCNJ3* mRNA expression was present only in tumour cells, but not in surrounding normal cells (figure 2D, middle and right). RNA ISH results correlated positively with IHC results (*p*<0.05, *r_S_*=0.625; figure 3B; see also table 1), and correlation with microarray data was highly significant (*p*<0.001, *r_S_*=0.861; figure 3C).

10.1136/jclinpath-2016-203798.supp5 Supplementary file

## Discussion

Here we report two methods for the detection of *KCNJ3* in archived FFPE human breast cancer tissue. Our study shows that accurate immunohistochemical detection of GIRK1 in human tissue is challenging due to low analytical specificity and/or sensitivity of available antibodies. Liang *et al*^7^ arrived at similar conclusions, aiming to assess GIRK1 and GIRK4 protein expression by immunofluorescence. While the anti-GIRK4 antibody used proved sufficiently specific and sensitive in their study, none of the anti-GIRK1 antibodies tested (comprising also Ab#5 evaluated in the current study) was suitable due to the high background staining in negative controls.^7^ This was supported by our finding that some samples with low *KCNJ3* mRNA expression still displayed elevated protein expression in IHC, leading to unconvincing correlation coefficients when IHC results were compared with microarray and ISH data. It should be stated at this point that discrepancies between microarray and IHC data might occur, since mRNA and protein expression levels might differ.^14^ However, our data point towards differences in performance and robustness of the detection methods used and not towards biological reasons for highly differential mRNA and protein expression. Remarkably, few studies succeeded in immunohistochemical staining of GIRK1 on FFPE human tissue using Ab#4^8^ and Ab#5.^3^ ^15^ ^16^ In retrospect, these previous studies might have overestimated GIRK1 expression, since anti-GIRK1 antibodies might lead to unspecific background staining. In addition to the IHC, we aimed to establish an antibody-independent method to detect *KCNJ3* expression in human FFPE tissue that would allow to visualise mRNA expression in situ. The RNA ISH technique described here offers both high specificity and sensitivity with virtually no background due to its specific probe design. This methodological design offers the possibility to reliably detect mRNA even in archived FFPE tissue with poor RNA quality.^17^ Evaluation of *KCNJ3* staining patterns demonstrated exclusive expression in neoplastic tumour epithelium, but not in the tumour stroma or in normal mammary parenchyma. The correlation of *KCNJ3* mRNA levels assessed by ISH with the corresponding microarray data was excellent (*r_S_*=0.861, *p*<0.001), indicating superiority of the RNA ISH method to IHC regarding robustness and reliability, even in FFPE tissue samples stored for up to 25 years. The ISH method presents a sensitive and specific detection technique that is an indispensable tool to efficiently investigate larger patient cohorts in order to derive possible prognostic and predictive information of *KCNJ3* in breast cancer. In general, ion channels are gaining increasing interest as new targets in cancer development and metastasis.^18^ Among these, potassium channels have shown to promote proliferation and apoptosis and to be involved in stemness of cancer cells.^19^ ^20^ Several studies showed an upregulation of *KCNJ3* in breast cancer, but its function in cancer cell behaviour needs to be further studied.^2^ ^3^ ^15^ We are confident that the comprehensive characterisation of *KCNJ3* detection techniques presented here will aid in further clarification of expression patterns, function and relevance of this intriguing cellular target in malignant diseases, and in physiological conditions.

**Take home messages**

- Establishment of GIRK1 immunohistochemistry is challenging due to low specificity and/or sensitivity of available anti-GIRK1 antibodies.
- RNA in situ hybridisation is superior to IHC in detecting KCNJ3 in human tissue.
- The KCNJ3 RNA ISH method presented here is robust, reliable, highly sensitive and specific.
