# Supplementary material for: Critical evaluation of KCNJ3 gene product detection in human breast cancer: mRNA in situ hybridisation is superior to immunohistochemistry
Source: J Clin Pathol. 2016 Oct 3;69(12):1116–21. doi: 10.1136/jclinpath-2016-203798 (PMC5256407; doi:10.1136/jclinpath-2016-203798)
Supplement: Supplementary file [file jclinpath-2016-203798supp1.pdf]

SUPPLEMENTARY FILE 1

Immunohistochemistry

HL-1, HEK-293, MCF-7 wild type and MCF-7 cells overexpressing GIRK1 were pelleted, formalin fixed and agarose-embedded for immunohistochemistry (IHC). FFPE mouse tissue was a gift from Ernst Malle (Institute of Molecular Biology and Biochemistry, Medical University of Graz). 3 µm thick FFPE sections were mounted on coated glass slides (Dako). On slides with FFPE sections of patient samples, FFPE HL-1 and HEK-293 cells were mounted in addition and served as positive and negative on slide controls. Slides were incubated at 60 °C for 1 hour or overnight, dewaxed and rehydrated. Staining conditions were optimized by systematic variation of staining conditions: Heat-induced epitope retrieval (HIER) was performed by incubating the sections either in a water bath (sodium citrate buffer pH 6.0) or alternatively in target retrieval solution pH 9.0 from Dako (Glostrup, Denmark; #S237584) for 40 minutes in a microwave at 150 W per cuvette (total volume 200 ml). The slides were cooled, rinsed in distilled water and incubated in either PBS or wash buffer from Dako for 5 min to achieve pH equilibration. Tissue areas to be stained were encircled with a hydrophobic barrier pen (Dako). Endogenous peroxidase activity was blocked by incubation with 3% hydrogen peroxide (H_2O_2; Dako) for 10 min. Slides were washed three times in either PBS or Dako wash buffer, followed by antibody incubation for 1 hour (Ab#1, diluted 1:50 or 1:100 in antibody diluent solution from Dako). Slides were subsequently washed as above and incubated with the EnVision+ dual link reagent (rabbit/mouse-HRP, from Dako). The chromogen diaminobenzidine (DAB; from Dako; 7 min incubation) was used for visualization, slides were rinsed in PBS or Dako wash buffer, counterstained with Meyer's hematoxylin, rinsed in hand warm tap water for 5 min, dehydrated, and cover slipped with Entellan® (Merck). Cytoplasmic staining intensity in tumor tissue was scored by two independent investigators (SWJ and TB) using a semi-quantitative four tiered score with 0-3 corresponding to no staining above background (0), weak (1), intermediate (2) and strong (3) staining. Incubation without primary antibody served as additional negative control.
