# Supplementary material for: Critical evaluation of KCNJ3 gene product detection in human breast cancer: mRNA in situ hybridisation is superior to immunohistochemistry
Source: J Clin Pathol. 2016 Oct 3;69(12):1116–21. doi: 10.1136/jclinpath-2016-203798 (PMC5256407; doi:10.1136/jclinpath-2016-203798)
Supplement: Supplementary file [file jclinpath-2016-203798supp2.pdf]

SUPPLEMENTARY FILE 2

Example of image analysis

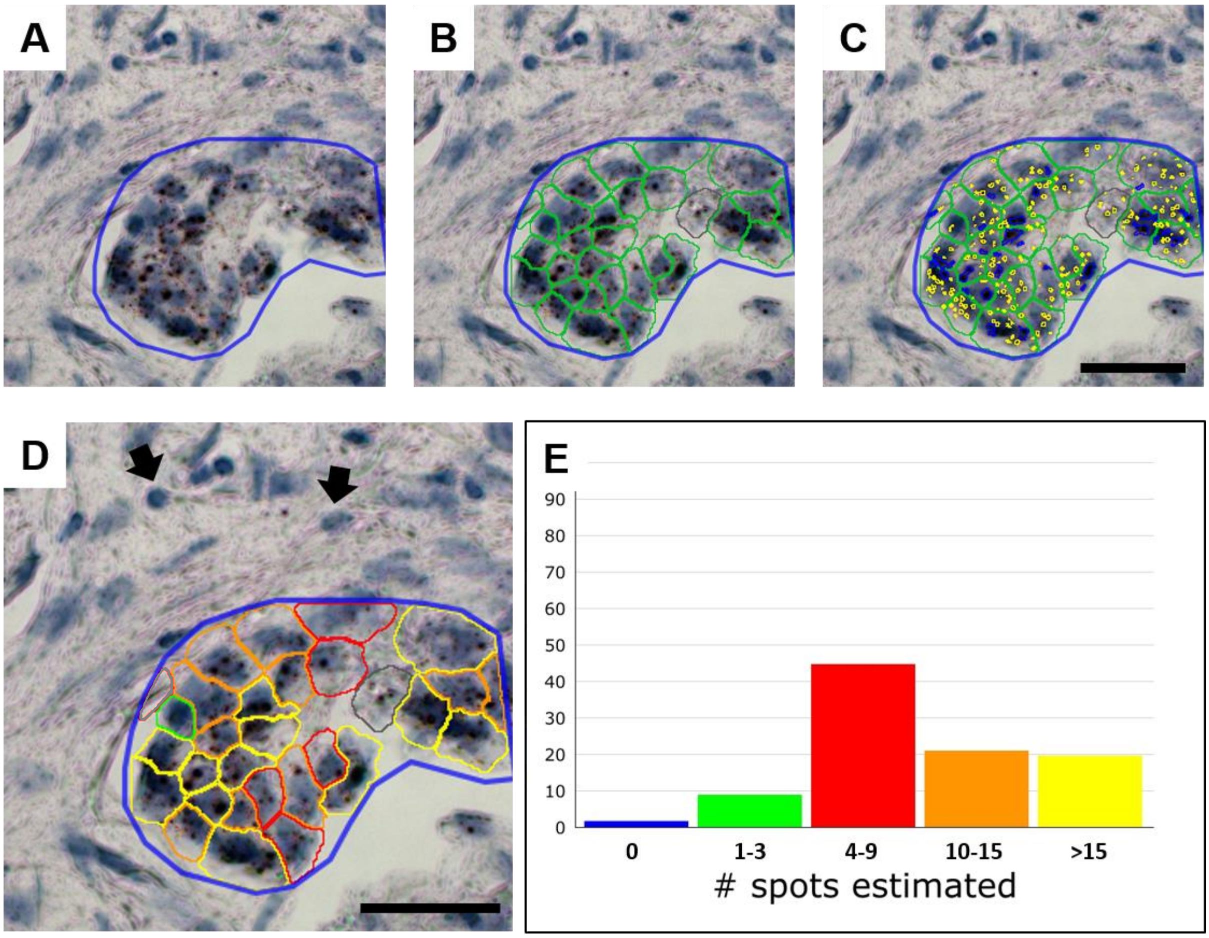

Figure S1: Detail of digital analysis of *KCNJ3* mRNA expression as assessed by RNA ISH in patient sample #2.

Single steps during image analysis include **A.** definition of the area of interest (blue), **B.** detection of single cells (green) and **C.** detection of single spots (yellow) and clusters (blue). **D.** Graphical visualization of results. Colours correspond to the bars in E, grey cells were excluded for analysis by manual quality control. **E.** Bar graph of final results. *The average number of spots estimated for this sample was 10.11. Scale bar: 25 µm in all images.*
