# Supplementary material for: Critical evaluation of KCNJ3 gene product detection in human breast cancer: mRNA in situ hybridisation is superior to immunohistochemistry
Source: J Clin Pathol. 2016 Oct 3;69(12):1116–21. doi: 10.1136/jclinpath-2016-203798 (PMC5256407; doi:10.1136/jclinpath-2016-203798)
Supplement: Supplementary file [file jclinpath-2016-203798supp3.pdf]

SUPPLEMENTARY FILE 3

Specificity and sensitivity of anti-GIRK1 antibodies on western blots.

Specificity and sensitivity of the different anti-GIRK1 antibodies was first assessed by Western blot analysis (methodological details are given below) using protein obtained from GIRK1/4-transfected and untransfected *Xenopus leavis* oocytes, HEK-293, HL-1 and MCF-7 cells. Antibody dilutions ranging from 1:100 to 1:1500 were used to determine the optimal concentration for each antibody (Figure S2). Shortly, four antibodies (Ab#1-3 and Ab#6) produced specific GIRK1 signals at 56 and 72 kD (representing non-glycosylated and glycosylated forms of GIRK1), but two of them (Ab#3 and Ab#6) at low intensities. Three antibodies (Ab#2-3 and Ab#5) produced additional bands not clearly attributable to GIRK1 in negative controls and one antibody (Ab#4) did not lead to any conclusive results. Incubation of membranes without primary antibodies resulted in clean lanes without any bands (data not shown). In summary, Western blot analysis recommended Ab#1 (Figure S2B) for best sensitivity and specificity and was therefore selected for further tests in immunohistochemistry (IHC).

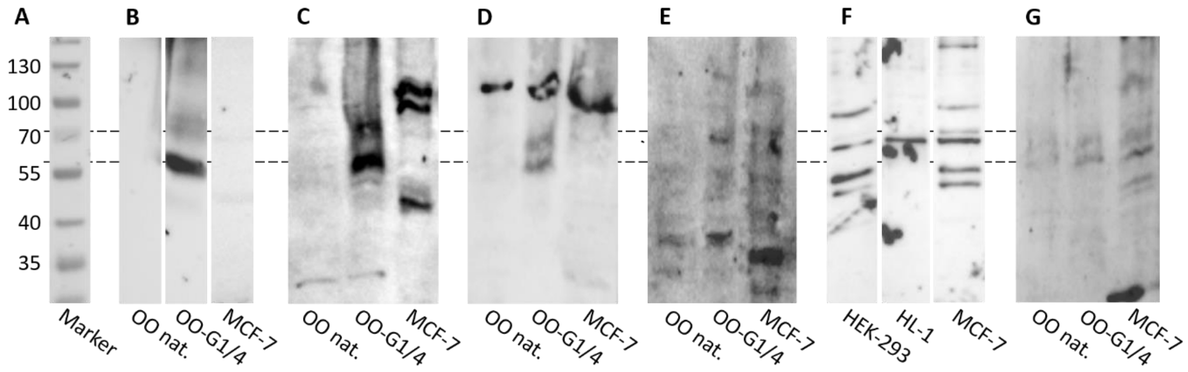

Figure S2: Specificity and sensitivity of anti-GIRK1 antibodies on Western blots.

A. Protein marker (molecular weight in kiloDalton is given to the left of each band).

B. Ab#1, diluted 1:1000, produced specific bands at 56 kD and 72 kD (representing non-glycosylated and glycosylated forms of GIRK1) at reasonable intensity in the positive control (OO-G1/4: *Xenopus laevis* oocytes overexpressing human GIRK1/GIRK4 heterotetrameric

channels. The negative control (native *X. laevis* oocytes) and MCF-7 breast cancer cells were negative. **C.** Ab#2, diluted 1:1000, produced specific bands at reasonable intensity in the positive control (OO-G1/4). The negative control (OO nat.) and MCF-7 cells resulted in additional bands that did not represent the expected band sizes for GIRK1. **D.** Ab#3, diluted 1:650, produced specific signals but at low intensity in the positive control (OO-G1/4), deemed unsuitable for sensitive GIRK1 protein detection in immunohistochemistry (IHC). In addition, strong bands at approx. 120 kD were present with equal intensity in all three lanes. **E.** Ab#4, diluted 1:200, did not display any bands that could be unequivocally attributed to GIRK1 protein expression and produced high background. **F.** Ab#5, diluted 1:200, might display a positive signal in the positive control (HL-1 cells), but resulted in additional bands in both the negative control (HEK-293) and in MCF-7 cells that could not be clearly attributed to GIRK1. *HEK-293 and HL-1 cells served as alternative negative and positive controls since they were used as controls also in IHC and X. laevis oocytes were not available.* **G.** Ab#6, diluted 1:200, produced specific bands but at low intensity in the positive control (OO-G1/4). MCF-7 cells resulted in additional bands that did not represent the expected band sizes for GIRK1.

30 µg total protein were loaded for oocytes, 75 µg protein for cell lines. The dashed line represents the expected locations of specific bands for GIRK1 in its native and glycosylated form at 56 and 72 kD.

Western blotting procedure

Xenopus leavis oocytes were prepared and transfected with GIRK1 and GIRK4 by cRNA injection as described previously [1]. Oocytes or mammalian cells (MCF-7, HL-1 and HEK-293) were harvested, washed, pelleted and lysed in radio immunoprecipitation assay (RIPA) buffer (Thermo Scientific) containing a protease inhibitor cocktail (PIC, Sigma-Aldrich). After centrifugation (13000*g* at 4 °C for 20 min), protein concentrations of the supernatants were determined using the bicinchoninic acid (BCA) protein assay kit (Thermo Scientific) according to manufacturer’s instructions. Aliquots (containing 30 µg protein for oocytes, 75 µg protein for

cell lines) were heat-denatured at 95 °C for 5 min, subjected to sodium dodecyl sulfate-polyacrylamide gel electrophoresis (SDS-PAGE; 8% gels) and electro-transferred onto a nitrocellulose membrane (GE Healthcare). The membranes were incubated for 1 hour in blocking solution (PBST containing 5% non-fat dry milk) followed by antibody incubation over night at 4 °C. Membranes incubated without primary antibody served as controls. The membranes were washed 3 times with PBST before incubation with the appropriate secondary antibodies for 1 hour. Detection of antibody binding was performed with the enhanced chemiluminescence (ECL) Select detection solution (GE Healthcare) followed by exposure to a CCD (charge-coupled device) camera using the ChemiDoc™ MP System (Bio-Rad).

References

1 Wagner, V., E. Stadelmeyer, M. Riederer*, et al*. Cloning and characterisation of GIRK1 variants resulting from alternative RNA editing of the KCNJ3 gene transcript in a human breast cancer cell line. J.Cell.Biochem. 2010;**110**:598-608.
