# Supplementary material for: Critical evaluation of KCNJ3 gene product detection in human breast cancer: mRNA in situ hybridisation is superior to immunohistochemistry
Source: J Clin Pathol. 2016 Oct 3;69(12):1116–21. doi: 10.1136/jclinpath-2016-203798 (PMC5256407; doi:10.1136/jclinpath-2016-203798)
Supplement: Supplementary file [file jclinpath-2016-203798supp4.pdf]

SUPPLEMENTARY FILE 4

Optimization of the GIRK1 IHC for FFPE human breast cancer samples

For further IHC protocol optimization with the chosen antibody, breast cancer patient sample #2 with high GIRK1 mRNA expression levels according to the microarray data was used as biological positive control. Samples incubated without primary antibody were used as technical negative controls and cell pellets from HEK-293 and HL-1 were used as on slide negative and positive controls, respectively (figure S3). We systematically tested different conditions regarding heat induced epitope retrieval (HIER; water bath with pH 6 versus microwave with pH 9), wash buffer (PBS versus commercial Dako wash buffer), antibody dilutions (1:50 versus 1:100), and their respective combinations. In summary, the combination of HIER at pH 9 (microwave), usage of Dako wash buffer and an antibody dilution of 1:50 of Ab#1 lead to best staining results with highest specificity and lowest background (figure S3, last row).

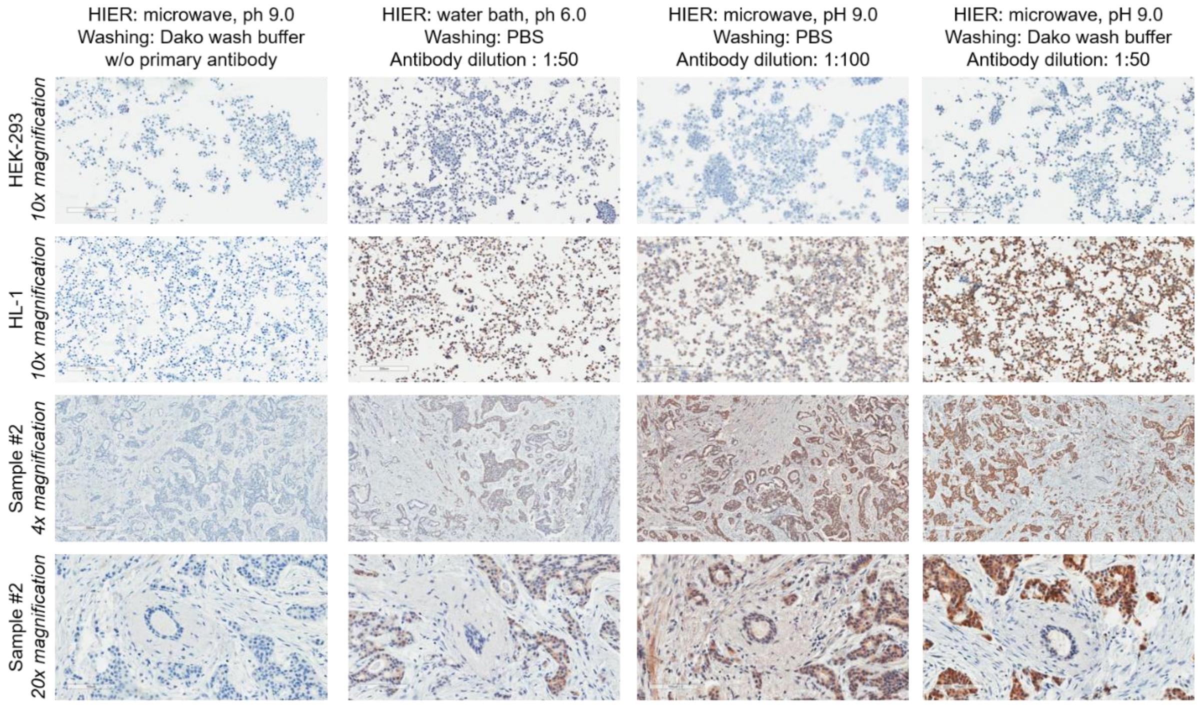

Figure S3: Optimization of the GIRK1 immunohistochemistry on a FFPE human breast cancer sample and on slide control cell pellets using Ab#1.

Different conditions regarding heat induced epitope retrieval (HIER), wash buffer, and antibody dilutions were tested as described on top of each row. HEK-293 cells (first line) and HL-1 cells (second line) served as negative and positive on slide controls, respectively. Micrographs of sample #2 were taken at two different magnifications as indicated (third and last line). Incubation without Ab#1 served as technical negative control (first row). HIER with pH 6 resulted in reduced staining intensity presumably due to incomplete antigen retrieval (second row). Slides rinsed with phosphate buffered saline (PBS) as wash buffer displayed significantly higher background staining compared to stains washed with DAKO wash buffer, even in conjunction with lower antibody concentrations (third row). Best results regarding signal-to-background ratio were obtained using the combination of HIER at pH 9, washing with Dako wash buffer and antibody dilution of 1:50 (last row). *PBS: phosphate buffered saline.*
