# Supplementary material for: Critical evaluation of KCNJ3 gene product detection in human breast cancer: mRNA in situ hybridisation is superior to immunohistochemistry
Source: J Clin Pathol. 2016 Oct 3;69(12):1116–21. doi: 10.1136/jclinpath-2016-203798 (PMC5256407; doi:10.1136/jclinpath-2016-203798)
Supplement: Supplementary file [file jclinpath-2016-203798supp5.pdf]

SUPPLEMENTARY FILE 5

Biological positive control used in each run of RNA ISH

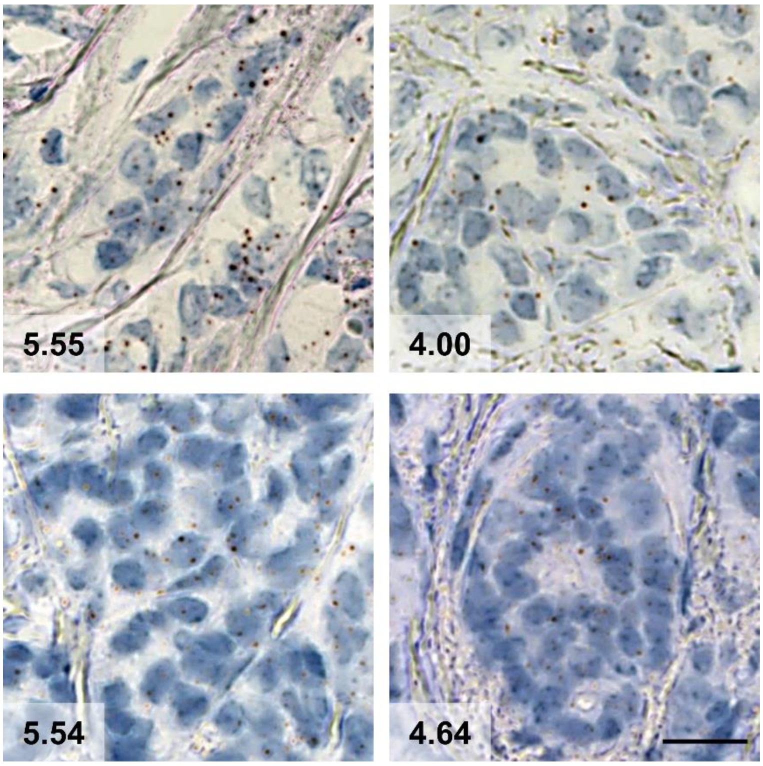

Figure S4: Reproducibility of RNA *in situ* hybridization.

Consecutive sections of the same patient sample (sample #6) were hybridized in different runs using the *POLR2A* probe (positive control). Numbers in boxes indicate the average number of spots per cell as determined with the SpotStudio software, and indicate that results were comparable between runs. Scale bar: 20 µm; all images shown at identical magnifications.
